# Metformin does not compromise energy status in human skeletal muscle at rest or during acute exercise: A randomised, crossover trial

**DOI:** 10.14814/phy2.14307

**Published:** 2019-12-12

**Authors:** Jonas M. Kristensen, Christian Lillelund, Rasmus Kjøbsted, Jesper B. Birk, Nicoline R. Andersen, Lars Nybo, Karin Mellberg, Anudharan Balendran, Erik A. Richter, Jørgen F. P. Wojtaszewski

**Affiliations:** ^1^ Section of Molecular Physiology Department of Nutrition, Exercise and Sports Faculty of Science University of Copenhagen Copenhagen Denmark; ^2^ The University Hospitals Centre for Health Research UCSF Copenhagen University Hospital Copenhagen Denmark; ^3^ Section of Integrative Physiology Department of Nutrition, Exercise and Sports Faculty of Science University of Copenhagen Copenhagen Denmark; ^4^ Astra Zeneca R&D Mölndal Gothenburg Sweden; ^5^ Present address: Laird Thermal Systems Gothenburg Sweden; ^6^ Present address: Alligator Bioscience AB Lund Sweden

**Keywords:** Akt, AMPK activity, exercise, insulin sensitivity, metformin content in muscle, TBC1D1, TBC1D4

## Abstract

5´AMP–activated protein kinase (AMPK) is a mediator of a healthy metabolic phenotype in skeletal muscle. Metformin may exacerbate the energy disturbances observed during exercise leading to enhanced AMPK activation, and these disturbances may provoke early muscular fatigue. We studied acute (1 day) and short‐term (4 days) effects of metformin treatment on AMPK and its downstream signaling network, in healthy human skeletal muscle and adipose tissue at rest and during exercise, by applying a randomized blinded crossover study design in 10 lean men. Muscle and fat biopsies were obtained before and after the treatment period at rest and after a single bout of exercise. Metformin treat ment elicited peak plasma and muscle metformin concentrations of 31 μM and 11 μM, respectively. Neither of the treatments affected AMPK activity in skeletal muscle and adipose at rest or during exercise. In contrast, whole‐body stress during exercise was elevated as indicated by increased plasma lactate and adrenaline concentrations as well as increased heart rate and rate of perceived exertion. Also whole‐body insulin sensitivity was enhanced by 4 days metformin treatment, that is reduced fasting plasma insulin and HOMA‐IR. In conclusion, acute and short‐term metformin treatment does not affect energy homeostasis and AMPK activation at rest or during exercise in skeletal muscle and adipose tissue of healthy subjects. However, metformin treatment is accompanied by slightly enhanced perceived exertion and whole‐body stress which may provoke a lesser desire for physical activity in the metformin‐treated patients.

## Graphical abstract

Acute or short‐term metformin treatment did not affect the energy status and 5´AMP–activated protein kinase activation at rest or during exercise in skeletal muscle and adipose tissue of healthy subject. However, the metformin treatment was accompanied by enhanced whole‐body stress.

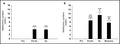

## 1 INTRODUCTION

Metformin is the most prevalent drug for treatment of type II diabetic patients and other patient groups with decreased insulin sensitivity (Boyle, Salt, & McKay, 2010). Metformin does not affect insulin secretion but improves insulin sensitivity, reflected by a decreased glucose production by the liver, and also by enhanced glucose uptake and oxidation in skeletal muscle and adipose tissue (Bailey, 2005; Stumvoll, Haring, & Matthaei, 2007).

The molecular mechanisms behind the effects of metformin are not fully clarified but inhibition of complex I in the mitochondrial electron transport chain has been reported in liver and muscle cells as well as skeletal muscle (Brunmair et al., 2004; Carvalho et al., 2008; El Mir et al., 2000; Owen, Doran, & Halestrap, 2000; Turner et al., 2008). Also activation of the 5´‐AMP‐activated protein kinase (AMPK) has been shown in skeletal muscle and adipose tissue after long‐term metformin treatment in diabetic patients (Boyle et al., 2011; Musi et al., 2002). However, in the liver the first studies suggesting the effects of metformin to be AMPK‐dependent (Shaw et al., 2005; Zhou et al., 2001) have recently been questioned by studies showing AMPK‐independent effects of metformin with regard to reduction of hepatic glucose production (Foretz et al., 2010; Hunter et al., 2018; Madiraju et al., 2018; Miller et al., 2013).

AMPK is a heterotrimeric kinase composed of a catalytic α subunit and regulatory β and γ subunits. In human skeletal muscle, three trimeric complexes have been identified (α1β2γ1, α2β2γ1, and α2β2γ3) (Wojtaszewski et al., 2005). AMPK activity is upregulated, both covalently by phosphorylation at Thr172 and allosterically by AMP/ADP binding, when the cellular energy balance is threatened, indicated by an increased AMP:ATP ratio (Towler & Hardie, 2007). In contrast to skeletal muscle, where it is a common finding that AMPK is activated during exercise (Jorgensen, Richter, & Wojtaszewski, 2006), only two studies have to our knowledge examined AMPK activation in human adipose tissue in relation to exercise (Kristensen et al., 2007; Watt et al., 2006). Watt et al. reported increased AMPK activity after 90 min cycle ergometer exercise whereas our laboratory did not observe AMPK activation after 40 min one‐legged knee extensor exercise with superimposed arm cranking to increase sympatho‐adrenal activity (Kristensen et al., 2007).

So whereas both long‐term metformin treatment and acute exercise per se have been verified as AMPK activators in skeletal muscle and adipose tissue, the *acute* effect of metformin treatment on AMPK activation in human skeletal muscle and adipose tissue at rest and during exercise has not been described. We hypothesized that acute metformin treatment increases AMPK activity and signaling in human skeletal muscle and adipose tissue. Furthermore, that metformin enhances the exercise‐induced activation of AMPK. To test this hypothesis we used 1 or 4 days metformin/placebo treatment of healthy subjects in combination with an acute bout of one‐legged knee extensor exercise.

## 2 METHODS

### 2.1 Ethical approval

Healthy, moderately trained men gave their informed consent to participate in an “acute” (A‐MES, *n* = 9) or a “short‐term” (ST‐MES, *n* = 10) metformin‐treatment study, respectively, each including one bout of one‐legged knee exercise. Studies were approved by the Copenhagen Ethics Committee (#H‐KF‐277313) and conformed with the declaration of Helsinki except for registration in a database. Peak pulmonary oxygen uptake (VO_2 peak_) was determined during an incremental cycle ergometer test and peak work load (PWL) of the knee extensors was determined in a modified Krogh bicycle ergometer that allows dynamic contractions of only the knee extensors (Table 1) (Andersen, Adams, Sjogaard, Thorboe, & Saltin, 1985).

**Table 1:** Characteristics for participants in the acute and the short‐term metformin exercise studies

	Acute study	Short‐term study
Age (years)	24 ± 1	24 ± 1
Weight (kg)	81.8 ± 1.9	76.2 ± 2.9
Height (m)	1.84 ± 0.02	1.86 ± 0.08
BMI (kg/m^2^)	24.2 ± 0.6	22.0 ± 0.6
VO_2 peak_ (ml O_2_ kg^−1^ min^−1^)	53.7 ± 1.2	52.3 ± 0.9
PWL (W)	50.0 ± 2.4	48.0 ± 2.0

Note

Data are means ± *SEM*. *n* = 9 in acute study; *n* = 10 in short‐term study.

Abbreviations: PWL, peak work load of the knee extensors determined on a modified bicycle ergometer during an exercise test with one leg; VO_2 peak_, peak pulmonary oxygen consumption determined on a bicycle ergometer using both legs.

### 2.2 Study protocols

#### 2.2.1 Acute metformin exercise study (A‐MES)

**Figure 1:**
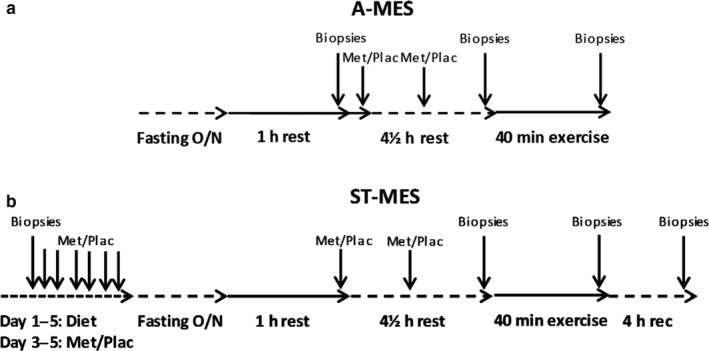
(a and b) Illustration of the experimental study designs for the A‐MES and the ST‐MES. A standardized meal was consumed together with the first Met/Plac dosage. A‐MES, acute metformin exercise study; ON, over night; ST‐MES, short‐term metformin exercise study

The study was conducted in a randomized single‐blinded crossover design in which each subject participated on two nearly identical experimental days. The only difference was that the subjects received metformin (Metformin Actavis, 500 mg, Actavis Group PTC) 1 day and placebo (Calcium: Dolomitkalk, Axellus A/S, Danmark) tablets the other day. The two experimental days were separated by 3 weeks. Subjects were asked to register their diet for 2 days prior to the first experimental day, and to eat a similar diet the 2 days preceding the second experimental day. Half of the subjects did the metformin trial first and the other half last (Figure 1a). The metformin treatment is within the recommended maximal clinical dose (Graham et al., 2011).

On the experimental day, the subject arrived in the morning after an overnight fast. After 1 hr of rest a blood sample was obtained and needle biopsies (termed “Pre”) from the vastus lateralis muscle in both legs and an abdominal subcutaneous fat tissue biopsy were obtained under local anesthesia. Thereafter 1.5 g metformin/placebo were ingested together with water and a standardized breakfast (oatmeal topped with raisins, with a portion size of ~1,800 kJ corresponding to 15% of individual daily calorie intake.) at time 0. The following 4½ hr were spent lying on a bed. After 2 hr a second metformin dose of 1.5 g metformin/placebo was ingested (i.e., total daily dose of 3 g metformin) with water. At 4½ hr a second needle biopsy from the vastus lateralis muscle in both legs and a subcutaneous fat tissue biopsy were obtained (termed “Pre Ex.”). Immediately after, the subject performed one‐legged knee extensor exercise for 40 min with an intensity of 80% PWL. We were aiming at having the highest levels of plasma metformin during the exercise period with this timing of the metformin dosage. A biopsy from the exercising leg was taken immediately after termination of the exercise period (within 20 s) and biopsies from the resting leg and adipose tissue were taken within 1–1½ min after exercise termination (termed “Ex.”). Blood samples were drawn and heart rate (Polar S720i Polar Electro), oxygen uptake, and respiratory exchange ratio (RER) were registered frequently in the resting period and during the exercise period. Skeletal muscle data from the exercising leg, but not the resting leg, are included in this paper.

#### 2.2.2 Short‐term metformin exercise study (ST‐MES)

The A‐MES was conducted and results were analyzed before commencing of the ST‐MES. The reason why we added the ST‐MES later was due to a possible insufficient muscle tissue accumulation of metformin in the A‐MES.

The ST‐MES study was similar to the A‐MES conducted with a single‐blinded crossover design in which each subject participated in two nearly identical experimental periods, receiving metformin in one experimental round and placebo in the other (Figure 1b). Half of the subjects did the metformin treatment first and the other half last. The two experimental periods lasted for 6 days and were separated by 3 weeks.

Common side effects of metformin treatment, particularly at initiation of the treatment are gastrointestinal discomfort and nausea (Cusi & DeFronzo, 1998). To ameliorate these side effects, and circumvent the influence of the side effect on dietary intake between the placebo and metformin trials, the metformin dose was gradually increased, the diet was controlled during the treatment periods and metformin/placebo was taken together with a meal.

The individual habitual caloric intake was registered by 3 days of detailed dietary registration (food type and amount). Based on this registration, the individual habitual daily caloric intake was calculated (in average ~12,000 kJ) and used to personalize the caloric intake during a 6 days controlled diet during each of the two experimental periods. The controlled diet was initiated 2 days prior to the metformin/placebo treatment to get the participants customized to the diet before commencing the metformin/placebo treatment. Thus, at “day 1” subjects were subjected to the standardized diet (15% protein, 30% fat and 55% carbohydrate) which continued to day 6 (“day 6” being the experimental exercise day). At “day 3” the subjects arrived in the morning after an overnight fast. A resting venous blood sample was obtained and needle biopsies (termed “Pre”) from the vastus lateralis muscle in both legs and an abdominal subcutaneous fat tissue biopsy were obtained under local anesthesia (Xylocain, Astra Zenecia). Thereafter ½ g metformin/placebo were ingested together with water and a standardized breakfast. Thereafter subjects went home and continued with the diet for the following 3 days (“days 3–5”) together with metformin/placebo treatment, ingesting an increasing dose with 1 g on “day 3,” 1½ g on “day 4,” and 2 g the “day 5.” At “day 6,” the experimental exercise day, subjects arrived in the morning after an overnight fast. At time 0 a blood sample was obtained and 1½ g metformin/placebo was ingested together with water and a standardized breakfast. Thereafter the experimental day was identical to the A‐MES, except that additional muscle and fat biopsies were included 4 hr after the exercise period (termed “Recovery”). Also rate of perceived exertion (RPE) was evaluated during the exercise period in the ST‐MES (Borg, 1970). Skeletal muscle data from the exercising leg, but not the resting leg, are included in this paper.

### 2.3 Pulmonary gas analysis

Expired air was collected in Douglas bags and the O_2_ and CO_2_ contents were analyzed as previously described (Kristensen et al., 2007). RER was calculated as VCO_2_/VO_2_ and was measured continuously during rest and recovery and a single time during exercise (at 20 min of exercise).

### 2.4 Analysis of blood and plasma metabolites, substrates, metformin, and hormones

In the A‐MES and ST‐MES, blood samples were collected before the meal and 1, 2, 2½, 3, 3½, 4, and 4½ hr after the meal. During exercise, blood samples were collected twice (at 20 and 40 min of exercise). In addition, in the ST‐MES one fasting blood sample was collected prior to the 4 days metformin/Placebo treatment and three times in the recovery period after exercise (½, 2, and 4 hr into recovery after exercise). Plasma metformin analyses were performed by Astra Zeneca (Mölndal, Sweden) with liquid chromatography‐tandem mass spectrometry (LC‐MSMS) with a lower detecting limit of 0.02 µM. Plasma adrenaline, noradrenaline, and insulin concentrations were analyzed as previously described (Kristensen et al., 2007). Plasma glucose concentration was analyzed on a blood‐gas analyser (ABL800 FLEX; Radiometer).

### 2.5 Muscle and fat tissue handling

The muscle and fat biopsies obtained were frozen in liquid nitrogen within 20 s and stored at −80°C. Potential surface blood on fat biopsies was cleared away by quick washing in ice‐cold physiological saline solution, blotted dry, and frozen in nitrogen. The frozen muscle biopsy specimens were freeze‐dried and dissected free of visible fat, blood and connective tissue, under microscopic examination, before further analyses were performed.

### 2.6 Muscle metformin

Freeze‐dried and dissected muscle biopsies were homogenized in ice‐cold buffer (1.26 mM CaCl_2_, 5.33 mM KCl, 0.44 mM KH_2_PO_4_, 0.50 mM MgCl_2_, 0.40 mM MgSO_4_, 138 mM NaCl, 4.17 mM NaHCO_3_, 0.34 mM Na_2_HPO_4_, 5.56 mM D‐glucose; pH 7.4 adjusted with 10 mM HEPES) 1:100 dw/vol by a tissue lyser at 30 Hz for 2 × 1 min (Tissue Lyser II, Qiagen Retsch). Muscle homogenates were rotated end‐over‐end at 4°C for 1 hr, after which they were centrifuged for 20 min at 16,000 *g*. The supernatants were harvested as the muscle lysate. Muscle metformin analyses on the lysates were done by Astra Zeneca with LC‐MSMS. Metformin concentration in muscle is given as µmol × (L muscle volume)^−1^. This is calculated by 3.16 as conversion factor between muscle dry weight and volume wet weight muscle, and subtraction of extracellular volumes of 8% and 17% in resting and exercised muscle, respectively (Sjogaard & Saltin, 1982).

### 2.7 Muscle Cr and PCr

Freeze‐dried and dissected muscle biopsy specimens were extracted with perchloric acid and neutralized. Creatine (Cr), phosphocreatine (PCr), and lactate contents were analyzed as previously described (Lowry & Passonneau, 1972).

### 2.8 Muscle glycogen

Muscle glycogen content was measured in muscle homogenates as glycosyl units after acid hydrolysis (Lowry & Passonneau, 1972).

### 2.9 AMPK activity

AMPK trimer specific activity was measured on immunoprecipitates from 300 μg of muscle lysate protein as described previously (Birk & Wojtaszewski, 2006). Antibodies were kindly donated by Prof Hardie, University of Dundee, Scotland, UK.

### 2.10 Muscle and fat processing, SDS‐PAGE, and Western blot analyses

Muscle and fat lysates, SDS‐PAGE, and Western blot analyses were prepared and performed as previously described (Kristensen et al., 2007, 2014). In short, protein content and phosphorylation of muscle or fat lysate proteins were measured on samples boiled in Laemmli buffer before being subjected to SDS‐PAGE on self‐cast Tris‐HCl polyacrylamide gels. Proteins were transferred to a PVDF membrane (Immobilon Transfer Membranes; Millipore) by semidry blotting. Membranes were blocked in a washing buffer (10 mM Tris‐base, 150 mM NaCl and 0.25% Tween 20) containing low fat milk protein (2%) or BSA (3%) and then probed with primary antibodies and appropriate secondary antibodies. Some membranes were stripped (buffer containing 100 mM 2‐mercaptoethanol, 2% SDS, and 62.5 mM Tris‐HCl). After checking for successful removal of the primary antibody, the membranes were re‐probed with a new primary antibody.

#### 2.10.1 Antibodies used for immunoblotting of protein content and specific phosphorylation sites

AMPK α subunits phosphorylation at Thr172 (Cell Signalling Technology Inc, #2531); AMPK α protein content in muscle and adipose was evaluated using anti α2 and α1‐AMPK antibodies, respectively (provided by Prof Hardie DG, University of Dundee, UK); acetyl‐CoA carboxylase (ACC) phosphorylation at ACC1 Ser80 and ACC2 Ser221 (Upstate Biotechnology, #07‐303). The ACC phosphospecific antibody is raised against a peptide corresponding to the sequence in rat ACC1 containing the Ser79 phosphorylation site, but the antibody also recognized the human ACC1 and ACC2 when phosphorylated most likely at the corresponding Ser80 and Ser221, respectively (Abu‐Elheiga, Almarza‐Ortega, Baldini & Wakil, 1997); ACC protein content (Dako, Streptavidin/HRP P0397); Akt2 protein content (Cell Signalling Technology Inc, #3063); Akt phosphorylation at Thr308 (Cell Signalling Technology Inc, #9275); Akt phosphorylation at Ser473 (Cell Signalling Technology Inc, #9271); TBC1D1 protein (provided by Prof Hardie DG, University of Dundee, UK); TBC1D1 phosphorylation at Ser237 (Millipore, #2061452); TBC1D1 phosphorylation at Ser700 – (Cell Signalling Technology Inc, #9271); TBC1D4 protein content (Abcam, #24469); TBC1D4 phosphorylation at Ser318 (Cell Signalling Technology Inc, #8619); TBC1D4 phosphorylation at Ser588 (Cell Signalling Technology Inc, #8730); TBC1D4 phosphorylation at Thr642 (Symansis, NZ #3028 P1).

Secondary HRP‐conjugated antibodies used were from Dako or Jackson Immunoresearch.

Immunoreactive bands were visualized with enhanced chemiluminescense (ECL, Millipore) and detected and quantified with the use of a coupled device image sensor and 1D software (2000 MM, Kodak) or ChemiDoc XRS + system (Bio‐Rad).

### 2.11 Statistics

Data were tested for normal distribution and equal variance. In some datasets simple transformations were used to obtain normal distribution and equal variance. HOMA, 2 hr glucose/insulin (G/I) ratio, Matsuda ISI (composite), and fasting insulin were evaluated by paired *t* test. RPE was evaluated by Wilcoxon signed‐ranked test. All other data were evaluated by two‐way ANOVA with repeated measures. Significant main effects and interactions in the ANOVA test were further analyzed by Student‐Newman–Keuls method as post hoc test. Differences between groups were considered statistically significant when *p* < .05. All data are expressed as means ± *SEM*. Statistical analyses were performed in SigmaStat 4.0 (Systat Software).

## 3 RESULTS

### 3.1 Short‐term metformin treatment enhances whole‐body insulin sensitivity

**Figure 2:**
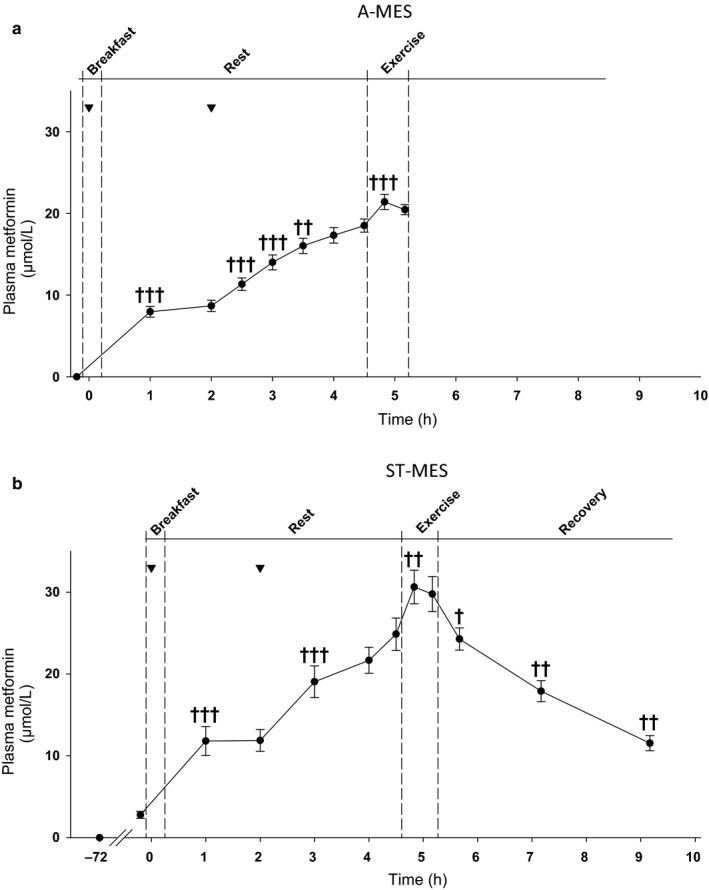
(a and b) Plasma metformin content in acute (a) and short‐term (b) metformin exercise trials. Data are means ± *SEM*. *n* = 9 in acute study; *n* = 10 in short‐term study. ^▼^: time for metformin dosage (1½ g). ^†^ *p* < .05, ^††^ *p* < .01, and ^†††^ *p* < .001 versus preceding value

*Plasma metformin concentration* after 3 days metformin treatment in the ST‐MES was ~3 µM (fasted overnight). Two hours after dosage of the first metformin dosage on the exercise experimental day, metformin concentrations were increased to ~9 and ~12 µM in the A‐MES and ST‐MES, respectively (Figure 2a,b). After dosing the second metformin dosage, at 2 hr, metformin concentrations increased during the following hours. Peak values of ~21 and 31 µM in the A‐MES and ST‐MES, respectively, were obtained during the exercise periods (Figure 2a,b).

*Plasma insulin* concentrations increased in all trials after the food intake, with peak values of ~150–200 pmol/L. Plasma insulin concentrations reversed to fasting levels within 4½ hr before initiating the exercise bout (Table 2). There was a generally lower insulin concentration during the metformin trial compared to placebo in the ST‐MES (main effect of trial *p* = .045, Table 2). Also fasting insulin (Table 3) and the HOMA‐IR index were lowered by ~34% and ~40%, respectively, with metformin treatment (Table 3) in the ST‐MES. In addition, 2 hr G/I ratio after a standardized meal was elevated with ~67% after metformin treatment in the ST‐MES, due to lowered insulin levels and no difference in glucose levels (Tables 2 and 3). The Matsuda ISI (composite) (DeFronzo & Matsuda, 2010; Matsuda & DeFronzo, 1999) in the ST‐MES study was 19.4 and 25.5 in the placebo and metformin trial, respectively (*p* < .05). Thus, the Matsuda ISI supports our data on the 2 hr G/I ratio, indicating an enhanced whole‐body insulin sensitivity with 4 days metformin treatment.

**Table 2:** Blood glucose and plasma insulin concentrations

Time (hr)	Glucose (mM)		Insulin (pmol/L)	
	Placebo	Metformin	Placebo	Metformin
Acute study				
0:00 (pre) diet	4.8 ± 0.1	4.9 ± 0.1	27 ± 4	28 ± 4
1:00	5.2 ± 0.3	4.9 ± 0.1	155 ± 25***	161 ± 26***
2:00	5.3 ± 0.2	5.0 ± 0.1	91 ± 13***†††	84 ± 16***†††
2:30	5.1 ± 0.2	4.9 ± 0.1	48 ± 10***†	86 ± 13***†
3:00	5.1 ± 0.2	4.7 ± 0.1	32 ± 3†††	42 ± 7†††
3:30	5.1 ± 0.2	4.9 ± 0.1	29 ± 5	32 ± 5
4:00	5.1 ± 0.1	4.8 ± 0.1	25 ± 2	35 ± 3
4:30	4.9 ± 0.1	4.7 ± 0.1	21 ± 3	29 ± 4
4:50 exercise	4.8 ± 0.1	4.7 ± 0.1	30 ± 4	32 ± 5
5:10 exercise	4.9 ± 0.1	4.8 ± 0.1	26 ± 3	20 ± 3
Short‐term study				
−72 (Pre)	5.0 ± 0.1	5.1 ± 0.1	27 ± 2	26 ± 2#
0:00 diet	4.9 ± 0.1	4.8 ± 0.1	31 ± 4	20 ± 2#
1:00	5.4 ± 0.4*††	5.6 ± 0.3*††	193 ± 25***†††	172 ± 14***†††#
2:00	4.7 ± 0.2	4.4 ± 0.2	111 ± 16***†††	61 ± 6***†††#
3:00	4.6 ± 0.2	4.6 ± 0.2	53 ± 10**†††	34 ± 4**†††#
4:00	4.8 ± 0.1	4.7 ± 0.1	28 ± 3††	24 ± 4††#
4:30	4.8 ± 0.2	4.7 ± 0.2	29 ± 5	24 ± 3#
4:50 exercise	4.9 ± 0.1	4.9 ± 0.1	33 ± 6	26 ± 3#
5:10 exercise	4.9 ± 0.1	5.0 ± 0.2	26 ± 3	20 ± 3#
5:40	4.9 ± 0.1	4.7 ± 0.2	18 ± 3	15 ± 3#
7:10	4.9 ± 0.1	4.8 ± 0.2	25 ± 5	18 ± 2#
9:10	4.8 ± 0.1	4.7 ± 0.2	22 ± 3	19 ± 4#

Note

Data are means ± *SEM*. *n* = 9 in acute study; *n* = 10 in short‐term study. A standardized meal was consumed at 0 hr together with placebo tablets or 1.5 g metformin. The second dose of metformin (1.5 g) or placebo was given at 2:00 hr.

a *p < .05, ∗∗*p* < .01, and ∗∗∗*p* < .001 versus “Pre”; ^†^ *p* < .05, ^††^ *p* < .01, and ^†††^ *p* < .001 versus preceding value; ^#^ *P* < .05 versus placebo trial (main effect).

**Table 3:** Whole‐body insulin sensitivity measurements

	Fasting insulin (μU/ml)		HOMA‐IR		2 hr g/I ratio	
	Placebo	Metformin	Placebo	Metformin	Placebo	Metformin
Acute study	3.9 ± 0.6	N.D.	0.8 ± 0.1	N.D.	9.7 ± 1.8	9.3 ± 1.4
Short‐term study	4.4 ± 0.5	2.9 ± 0.3*	1.0 ± 0.1	0.60 ± 0.1*	6.0 ± 0.6	10.0 ± 1.1*, †

Note

Data are means ± *SEM*. *n* = 9 in acute study; *n* = 10 in short‐term study. Glucose concentrations are expressed in mg/dl and insulin in µU/ml in the calculations presented in the table.

Abbreviations: 2 hr G/I, glucose/insulin ratio 2 hr after a standardized meal; N.D., not determined.

* *p* < .05 versus placebo trial.

*Blood glucose* concentrations, in the fasting state or after a standardized meal, were not affected by the metformin treatments in either of the two trials (Table 2).

### 3.2 Metformin treatment induces an increased whole‐body stress

*Blood lactate* concentrations were higher in the metformin compared to placebo trial at rest (from 3 hr) and during the exercise period in the A‐MES. In the ST‐MES, lactate concentrations were higher at rest (from 2 hr), during exercise and in recovery (2 hr into the exercise recovery period) in the metformin trial. Lactate concentrations increased to ~2.0 mM during the exercise periods in the A‐MES and ST‐MES placebo trials, and were higher, ~3.0 mM, in both metformin trials (Table 4).

**Table 4:** Blood lactate, plasma adrenaline, and noradrenaline

Time (hr)	Lactate (mM)		Adrenaline (nM)		Noradrenaline (nM)	
	Placebo	Metformin	Placebo	Metformin	Placebo	Metformin
Acute study						
0:00 (pre) diet	0.7 ± 0.1	0.6 ± 0.1#	0.08 ± 0.02	0.05 ± 0.01	0.95 ± 0.16	0.77 ± 0.12
1:00	1.2 ± 0.1***	1.1 ± 0.1***	0.05 ± 0.01	0.04 ± 0.01	1.07 ± 0.26	0.68 ± 0.11
2:00	0.9 ± 0.0††	0.9 ± 0.1**	N.D.	N.D.	N.D.	N.D.
2:30	0.8 ± 0.0	0.8 ± 0.1**	0.06 ± 0.01	0.04 ± 0.01	0.60 ± 0.1	0.67 ± 0.13
3:00	0.6 ± 0.0	0.9 ± 0.1**###	N.D.	N.D.	N.D.	N.D.
3:30	0.6 ± 0.0	0.9 ± 0.1**###	0.04 ± 0.01	0.05 ± 0.02	0.66 ± 0.15	0.67 ± 0.10
4:00	0.6 ± 0.0	0.9 ± 0.1**###	N.D.	N.D.	N.D.	N.D.
4:30	0.7 ± 0.0	0.9 ± 0.1**###	0.08 ± 0.04	0.10 ± 0.03	0.68 ± 0.17	0.69 ± 0.09
4:50 exercise	2.3 ± 0.2***†††	3.0 ± 0.3***†††###	0.28 ± 0.05***†††	0.35 ± 0.08***†††	2.45 ± 0.34***†††	2.50 ± 0.38***†††
5:10 exercise	2.2 ± 0.2***†	2.9 ± 0.4***	0.25 ± 0.05***	0.36 ± 0.07***	2.50 ± 0.24***	2.74 ± 0.32***
Short‐term study						
−72 (pre)	0.7 ± 0.0	0.7 ± 0.0	0.21 ± 0.04	0.26 ± 0.08	1.04 ± 0.23	1.16 ± 0.23
0:00 diet	0.7 ± 0.1	0.8 ± 0.0	0.19 ± 0.04	0.21 ± 0.04	0.93 ± 0.20	1.11 ± 0.32
1:00	1.4 ± 0.1***†††	1.5 ± 0.1***†††	N.D.	N.D.	N.D.	N.D.
2:00	0.9 ± 0.1††	1.2 ± 0.1***#	N.D.	N.D.	N.D.	N.D.
3:00	0.7 ± 0.0	1.2 ± 0.1***###	0.20 ± 0.05	0.19 ± 0.05	0.77 ± 0.15	1.33 ± 0.60
4:00	0.7 ± 0.1	1.4 ± 0.1***###	N.D.	N.D.	N.D.	N.D.
4:30	1.0 ± 0.1	1.7 ± 0.2***###	0.21 ± 0.08	0.34 ± 0.06##	1.17 ± 0.34	1.41 ± 0.33
4:50 exercise	2.5 ± 0.4***†††	3.4 ± 0.4***†††#	0.76 ± 0.14***†††	1.00 ± 0.17***†††#	2.40 ± 0.41***†††	2.58 ± 0.37***†††
5:10 exercise	2.3 ± 0.4***	3.0 ± 0.4***#	0.84 ± 0.13***	1.00 ± 0.17***	2.35 ± 0.54***	2.66 ± 0.46***
5:40	1.0 ± 0.1*†††	1.7 ± 0.2***†††###	0.29 ± 0.06†††	0.24 ± 0.04†††	1.03 ± 0.14†††	0.91 ± 0.22†††
7:10	0.8 ± 0.1	1.1 ± 0.1**†###	0.20 ± 0.03	0.20 ± 0.03	1.07 ± 0.15	0.76 ± 0.22
9:10	0.8 ± 0.1	1.0 ± 0.1*	0.17 ± 0.04	0.20 ± 0.05	1.29 ± 0.21	0.95 ± 0.17

Note

Data are means ± *SEM*. *n* = 9 in acute study; *n* = 10 in short‐term study. A standardized meal was consumed at 0 hr together with placebo tablets or 1.5 g metformin. The second dose of metformin (1.5 g) or placebo was given at 2:00 hr.

Abbreviation: N.D., not determined.

a *p < .05, ∗∗*p* < .01, and ∗∗∗*p* < .001 versus “Pre”; ^†^ *p* < .05, ^††^ *p* < .01, and ^†††^ *p* < .001 versus preceding value; ^#^ *p* < .05, ^##^ *p* < .01, and ^###^ *p* < .001 versus placebo trial.

*Plasma adrenaline* concentrations increased ~fourfold in the exercise periods and were higher immediately before commencing exercise and during the first 20 min of the exercise period in the ST‐MES metformin trial compared to placebo (Table 4). Plasma noradrenaline concentrations followed the same pattern as adrenaline with increases of ~threefold during the exercise period in all trials, but without any significant effect of metformin in either of the studies (Table 4).

*Rate of perceived exertion* was borderline significantly (*p* = .055) higher during exercise in the metformin trial compared to placebo in the ST‐MES (Table 5).

**Table 5:** Metabolic characteristics

	Oxygen uptake (L O_2_ min^−1^)		RER		Heart rate (bpm)		RPE (6–20)	
	Placebo	Metformin	Placebo	Metformin	Placebo	Metformin	Placebo	Metformin
Acute study								
Rest	0.3 ± 0.0	0.3 ± 0.0	0.89 ± 0.04	0.87 ± 0.05	65 ± 2	64 ± 1	N.D.	N.D.
Exercise	1.1 ± 0.1*	1.2 ± 0.1*	0.95 ± 0.05	0.96 ± 0.07	108 ± 6*	109 ± 5*	N.D.	N.D.
Short‐term study								
Rest	0.3 ± 0.0	0.3 ± 0.0	0.94 ± 0.06	0.92 ± 0.04	57 ± 1	60 ± 2‡	N.D.	N.D.
Exercise	1.1 ± 0.1*	1.1 ± 0.0*	0.88 ± 0.02	0.89 ± 0.02	115 ± 5*	121 ± 5*, ‡	14 ± 0	15 ± 1§
Recovery	0.3 ± 0.0†	0.3 ± 0.0†	0.83 ± 0.04	0.79 ± 0.02	61 ± 2†	65 ± 2†, ‡	N.D.	N.D.

Note

Data are means ± *SEM*. *n* = 9 in acute study; *n* = 10 in short‐term study.

bpm, beats per minute; N.D., not determined; RER, respiratory exchange ratio; RPE, rate of perceived exertion.

* *p* < .001 versus “Rest”;

^†^ *p* < .001 versus “Exercise”;

^‡^ *p* < .05 versus placebo trial;

^§^ *p* = .055 versus placebo trial.

#### 3.2.1 Pulmonary

*Oxygen uptake* increased ~three to fourfold during exercise compared to rest in both A‐MES and ST‐MES, but there were no effects of metformin treatment (Table 5).

*Heart rate* increased ~onefold during exercise in all trials, and there was a main effect of trial during the day with significant elevated heart rate in the metformin compared to placebo trial in the ST‐MES (Table 5).

*RER*, a measure of whole‐body substrate utilization, was not affected by exercise or metformin either in the A‐MES or ST‐MES (Table 5).

### 3.3 Muscle metformin content does not affect muscle metabolites

**Figure 3:**
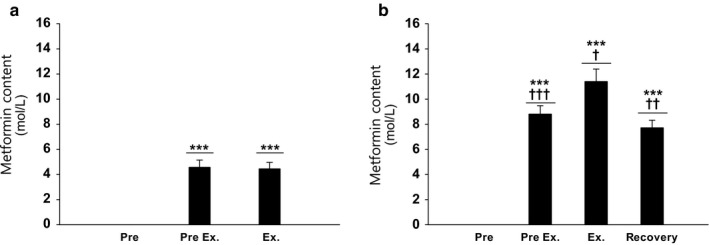
(a and b) Muscle metformin content for the exercising leg in the acute (a) and short‐term (b) metformin trials. Data are means ± *SEM*. *n* = 9 in acute study; *n* = 10 in short‐term study. ∗∗∗*p* < .001 versus “Pre”; ^†^ *p* < .05, ^††^ *p* < .01, and ^†††^ *p* < .001 versus preceding value

*Muscle metformin* concentration in the exercising leg at “Pre exercise,” that is 4½ hr after the first dosage of metformin on the experimental days, were ~4.5 µM in the A‐MES and ~9 µM in the ST‐MES (Figure 3a,b). Metformin content increased during exercise to ~11.5 µM in the ST‐MES (Figure 3b). Four hours into exercise “Recovery” metformin content was decreased to similar levels as “Pre exercise” in the ST‐MES (Figure 3b).

*Muscle Cr and PCr* levels increased and decreased, respectively, in the muscle of the working legs during exercise with no differences between placebo and metformin trials in the A‐MES and ST‐MES (Table 6). Consequently, the CrP/(Cr + CrP) ratio in the working legs decreased equally (~30%) in all trials during exercise (Table 6).

**Table 6:** Muscle substrates and metabolites

	Pre		Pre exercise		Post exercise		Recovery	
	Placebo	Metformin	Placebo	Metformin	Placebo	Metformin	Placebo	Metformin
Acute study exercising leg								
Creatine (Cr) (mmol (kg d.w.)^−1^)	55 ± 8	61 ± 4	56 ± 9	51 ± 5	80 ± 7***††	92 ± 15***††		
Phosphocreatine (PCr) (mmol (kg d.w.)^−1^)	91 ± 7	81 ± 6	89 ± 5	89 ± 5	61 ± 7**††	60 ± 8**††		
CrP/(Cr + CrP) ratio (×100)	63 ± 4	57 ± 2	63 ± 5	64 + 2	45 ± 4***†††	42 ± 7***†††		
Glycogen (mmol (kg d.w.)^−1^)	424 ± 16	425 ± 23	409 ± 18	430 ± 29	264 ± 34***†††	271 ± 49***†††		
Short‐term study exercising leg								
Creatine (Cr) (mmol (kg d.w.)^−1^)	65 ± 6	65 + 3	61 ± 4	68.1 ± 4.7	86 ± 5***†††	96 ± 6***†††	69 ± 5***	72 ± 6***
Phosphocreatine (PCr) (mmol (kg d.w)^−1^)	72 ± 7	73 ± 5	67 ± 5	69.3 ± 3.2	52 ± 8***††	52 ± 7***††	67 ± 5**	74 ± 5**
CrP/(Cr + CrP) ratio (×100)	52 ± 5	53 ± 2	52 ± 2	50.8 ± 2.0	36 ± 5***†††	35 ± 4***†††	50 ± 3†††>	51 ± 3†††
Glycogen (mmol (kg d.w.)^−1^)	478 ± 25	514 ± 21	537 ± 33	490 ± 33	312 ± 48***†††	300 ± 36***†††	343 ± 50***	296 ± 31***
Lactate (mmol (kg d.w.)^−1^)	10 ± 2	11 ± 1	11 ± 1	12 ± 2	20 ± 6**††	24 ± 6**††	10 ± 1††	10 ± 1††

Note

Data are means ± *SEM*. *n* = 9 in acute study; *n* = 10 in short‐term study.

a **p < .01 and ∗∗∗*p* < .001 versus “Pre”; ^††^ *p* < .01 and ^†††^ *p* < .001 versus preceding value.

*Muscle lactate* increased by ~80% in the working legs during exercise with no difference between trials in the ST‐MES (Table 6).

*Muscle glycogen* content was decreased (~40%) to the same extent during exercise in the working leg in all trials (Table 6).

### 3.4 AMPK is not regulated by acute or short‐term metformin treatment in skeletal muscle and subcutaneous adipose tissue

**Figure 4:**
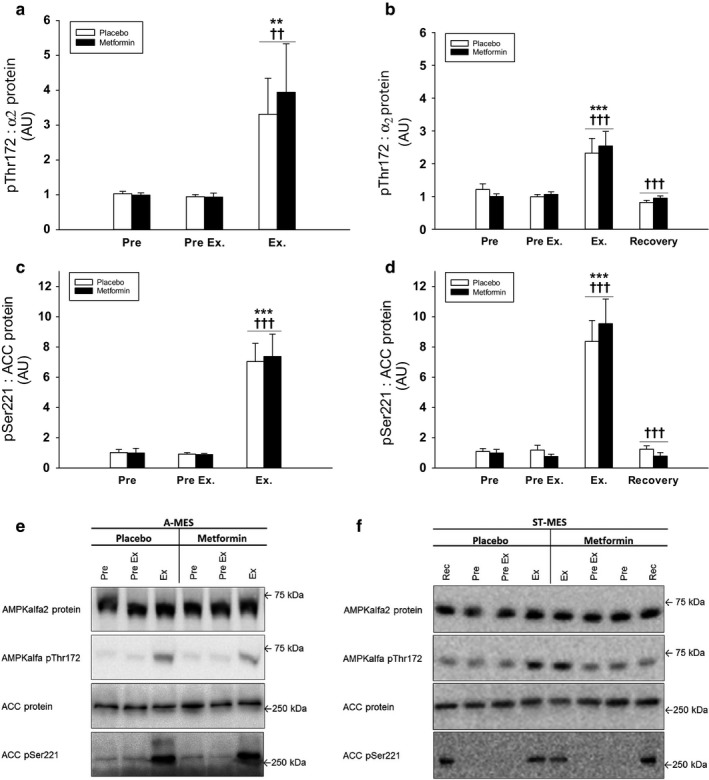
(a–f) Muscle AMPK pThr172: AMPKα2 protein and ACC pSer221: ACC protein in acute (a and c) and short‐term (b and d) metformin exercise studies. Data are means ± *SEM*. *n* = 9 in acute study; *n* = 10 in short‐term study. Black bars shows results for the metformin trial in the working leg. White bars shows results for the placebo trial in the working leg. Representative immunoblots are shown in figure (e and f). ∗∗*p* < .01 and ∗∗∗*p* < .001 versus “Pre”; ^††^ *p* < .01 and ^†††^ *p* < .001 versus preceding value. ACC, acetyl‐CoA carboxylase; AMPK, 5´AMP–activated protein kinase

*α‐AMPK Thr172 phosphorylation* increased during exercise in the working leg in all trials, with no difference between metformin and placebo trials (Figure 4a,b).

*ACC Ser221 phosphorylation*, an AMPK downstream target and a supposed marker of endogenous AMPK activity, including both covalent and allosteric activation, increased during exercise in the working leg in all trials, with no difference between metformin and placebo trials (Figure 4c,d). α2‐AMPK and ACC protein content were constant during the treatment and exercise periods in the working legs in all trials (data not shown).

**Figure 5:**
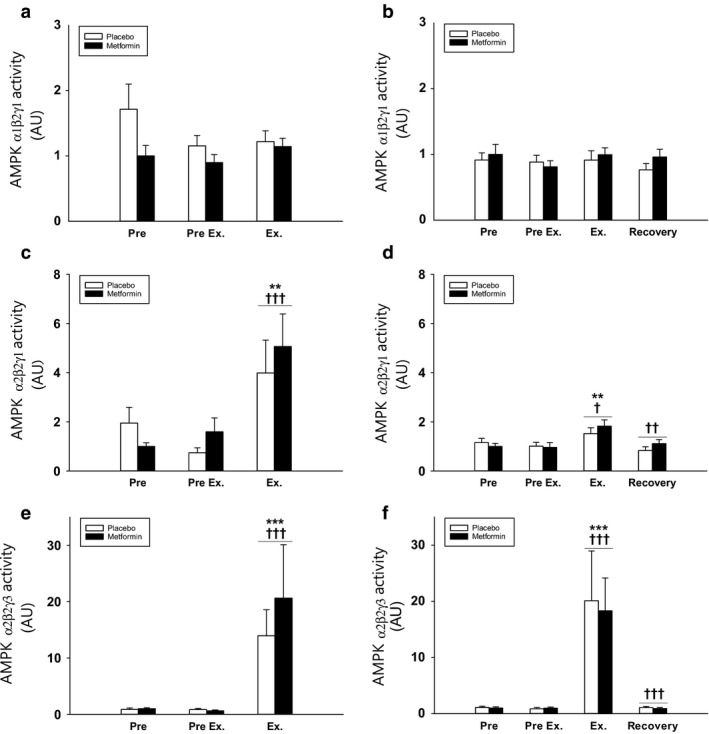
(a–f) Muscle AMPK trimercomplex activities in acute (a, c, and e) and short‐term (b, d, and f) metformin exercise studies. Data are means ± *SEM*. *n* = 9 in acute study; *n* = 10 in short‐term study. Black bars shows results for the metformin trial in the working leg. White bars shows results for the placebo trial in the working leg. ∗∗*p* < .01 and ∗∗∗*p* < .001 versus “Pre”; ^†^ *p* < .05, ^††^ *p* < .01, and ^†††^ *p* < .001 versus preceding value. AMPK, 5´AMP–activated protein kinase

*AMPK activity* of the α1β2γ1 complex activity was not affected by treatment or exercise in the A‐MES or ST‐MES (Figure 5a,b). AMPK α2β2γ1 and α2β2γ3 activities increased during exercise in the working leg in both the A‐MES and ST‐MES (Figure 5c–f). Neither resting complex activities nor the exercise‐induced activities were affected by metformin treatment compared to placebo in all trials (Figure 5c–f).

**Figure 6:**
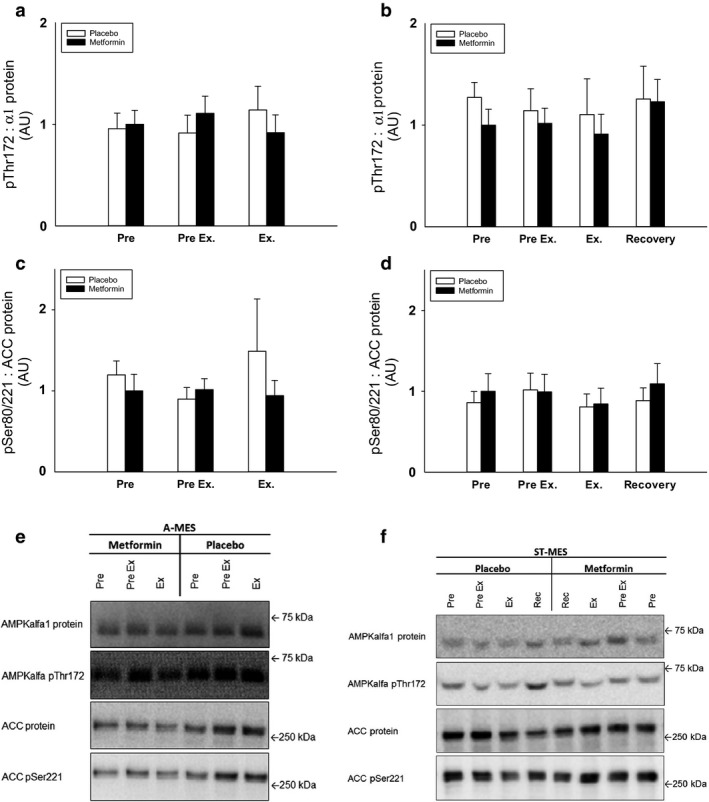
(a–f) Adipose AMPK pThr172: AMPKα1 protein and ACC pSer80/221:ACC protein ratio in acute (a and c) and short‐term (b and d) metformin exercise studies. Data are means ± *SEM*. *n* = 9 in acute study; *n* = 10 in short‐term study. Black bars shows results for the metformin trial. White bars shows results for the placebo trial. Representative immunoblots are shown in figure (e and f). ACC, acetyl‐CoA carboxylase; AMPK, 5´AMP–activated protein kinase

*α‐AMPK Thr172 phosphorylation and ACC Ser 80/221 phosphorylation* in the abdominal subcutaneous adipose tissue were unaffected by exercise and metformin treatment in all trials (Figure 6a–d). Also α1‐AMPK and ACC protein content were not different between trials in the A‐MES or ST‐MES (data not shown).

### 3.5 Metformin treatment does not affect AMPK and Akt2 signaling network at TBC1D1 and TBC1D4 in skeletal muscle

**Figure 7:**
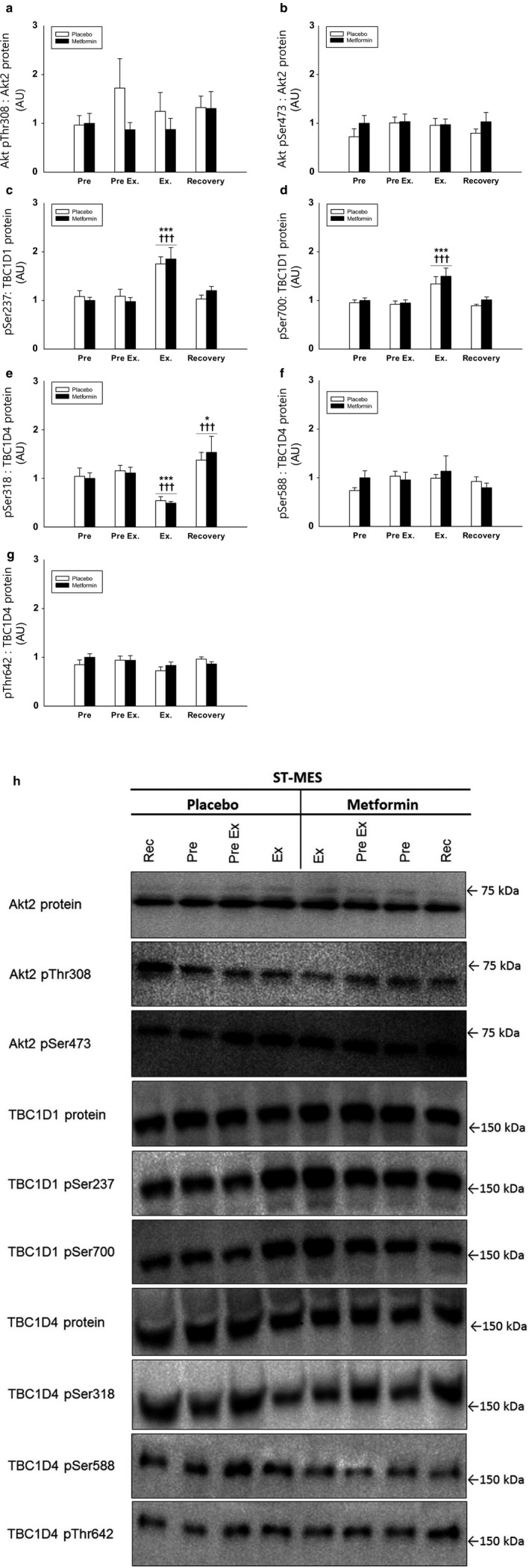
(a–c) Muscle Akt pSer308: Akt2 protein (a), and Akt pThr473: Akt2 protein (b), TBC1D1 pSer237: TBC1D1 protein (c), TBC1D1 pSer700: TBC1D1 protein (d), TBC1D4 pSer318: TBC1D4 protein (e), TBC1D4 pSer588: TBC1D4 protein (f) and TBC1D4 pThr642: TBC1D4 protein (g) in the short‐term study. Data are means ± *SEM*. Black bars shows results for the metformin trial. White bars shows results for the placebo trial. Representative immunoblots are shown in figure (h). ∗*p* < .05 and ∗∗∗*p* < .001 versus “Pre”; ^†††^ *p* < .001 versus preceding value

*Phosphorylation of Akt Thr308 and Ser473* (Figure 7a,b) *and Akt 2 protein* *(data not shown)* were not regulated by metformin and exercise per se or in combination.

*TBC1D1 Ser237 and Ser700 phosphorylation* (Figure 7c,d), both supposed AMPK targets, increased during exercise and decreased to baseline values in recovery. Neither of the sites was regulated by metformin treatment per se or in combination with exercise.

*Phosphorylation of TBC1D4 Ser318*, *Ser588, and Thr642* (Figure 7e–g), all Akt downstream targets, was not regulated by metformin per se. Phosphorylation of Ser318 decreased during exercise and increased in recovery, independent of metformin treatment.

TB1D1 and TBC1D4 protein abundance were not regulated by metformin and exercise per se or in combination (data not shown).

## 4 DISCUSSION

In the present study we investigated whether acute (A‐MES) or short‐term metformin treatment (ST‐MES) per se activates AMPK in skeletal muscle and abdominal subcutaneous adipose tissue, and whether metformin treatment potentiates AMPK activation during exercise.

In contrast to our hypothesis, metformin treatment did not affect AMPK activity in skeletal muscle or adipose. However, whole‐body stress during exercise was elevated, as indicated by increased adrenalin concentrations as well as increased heart rate and RPE.

Metformin is absorbed into tissues by different transport proteins (i.e., OCT 1 and OCT3). In mice up to seven times higher metformin concentrations have been reported to be accumulated in tissues as kidneys, pancreas, liver, and skeletal muscle compared to plasma levels (Graham et al., 2011; Wilcock & Bailey, 1994). We speculated that an explanation for missing increased AMPK activation following the A‐MES could be because of too short a period, that is 4–5 hr, for metformin to enter and accumulate in skeletal muscle and adipose cells, as well as the mitochondria compartments (Davidoff, 1971; Owen et al., 2000; Wilcock & Bailey, 1994; Zhou et al., 2001). Therefore, we added the ST‐MES to our investigation. Assuming AMPK activation by metformin is indirect through the inhibition of mitochondria complex I, the drug has to be transported from the cytosol into the mitochondria (Owen et al., 2000). The metformin values from the ST‐MES versus the A‐MES support our thoughts about tissue metformin accumulation. Thus, the exact identical metformin treatment on the exercise experimental days elicited substantially higher blood metformin concentrations in the ST‐MES compared to the A‐MES (AUC was ~45% higher in the ST‐MES compared to A‐MES during the exercise day) and the difference in muscle metformin concentration was even more pronounced with doubling of the content in the ST‐MES compared to A‐MES. The metformin contents in the blood, with peak values of ~20–30 µM, are in the upper level compared to reported clinical values (Cusi & DeFronzo, 1998). Recently Gormsen et al. (2016), using in vivo imaging of labeled ^11^C‐metformin by PET, reported a low metformin uptake rate but accumulation over time. This is in line with our observation of increasing metformin muscle content in skeletal muscle over time, that is elevated in ST‐MES compared to A‐MES. To the best of our knowledge we are the first to report metformin concentration in human skeletal muscle. Thus it is not possible to deduce if the muscle concentrations of ~5 and 11 µM obtained in the A‐MES and ST‐MES, respectively, are similar to the values in muscles from chronic metformin treated diabetic patients. Hence, in contrast to reports from human studies showing increased AMPK activity after long‐term (1–12 months) treatment (Boyle et al., 2011; Musi et al., 2002), it is difficult to infer whether our lack of an *acute and short‐term* metformin effect on AMPK activation is due to insufficient tissue and mitochondrial metformin accumulation.

Another possible explanation for the lack of an AMPK effect in the present study could be that healthy men respond differently to metformin treatment than insulin resistant and diabetic subjects. However, the study on insulin‐resistant individuals by Sharoff et al. (2010) also lacked AMPK activation in skeletal muscle despite 3 weeks metformin treatment. Moreover we have previously shown no difference in AMPK expression or basal and exercise‐induced activity in skeletal muscles from diabetics versus healthy controls (Kjobsted et al., 2016), although one study reported decreased exercise‐induced AMPK activation (Sriwijitkamol et al., 2007). Therefore, we assume that the subject characteristics are not the main explanation for the different effect of metformin in skeletal muscle between the present acute and the former chronic metformin study (Musi et al., 2002). Still, we cannot exclude that our lack of a metformin effect in the adipose tissue could be ascribed to body composition. Metformin effects have been shown to be positively related to BMI (Moreno‐Navarrete et al., 2011; Shikata et al., 2007) and the subjects in the present study were lean (BMI of 22–24) compared with the diabetic patients (BMI of 31) in the study by Boyle et al. (2011), reporting increased AMPK activation in adipose tissue after 10 weeks metformin treatment. Body region differences might also influence the results as Boyle et al. (2011) examined gluteal adipose tissue while we used abdominal subcutaneous biopsies in the present study. A limitation in interpretation of our study results is that only men were included. Thus, it cannot be excluded that women respond differently, although the results in the study by Sharoff et al. (2010) do not suggest a gender difference.

Our finding that fasting plasma lactate was unaffected by the 3 days metformin treatment in the ST‐MES is in accordance with the reports after short or long‐term metformin treatment (Gudat, Convent, & Heinemann, 1997; Johnson et al., 2007; Musi et al., 2002; Sharoff et al., 2010). However, the acute metformin dose on the experimental days in both the A‐MES and ST‐MES induced both higher resting and exercised induced plasma lactate compared to placebo. To our knowledge only a single study in addition to the present has reported a metformin effect on resting plasma lactate content (Boule et al., 2011) while some (Boule et al., 2011; Malin et al., 2010; Sharoff et al., 2010) but not all (Cunha et al., 2007, 2008; Fletcher, Hirji, Kuhn, Alexander, & Mucklow, 1986; Gudat et al., 1997) studies have observed an effect in combination with exercise. Although muscle lactate content increased during exercise, this increase was similar in the metformin and placebo trials, suggesting that the metformin‐induced increase in plasma lactate is not of muscle origin. Instead of muscle‐derived lactate, the liver could be the source for the increase in plasma lactate, given that gluconeogenesis from lactate has been shown to be reduced by metformin (Radziuk, Zhang, Wiernsperger, & Pye, 1997) although it is not a consistent observation in humans (Cusi, Consoli, & DeFronzo, 1996). Also increased lactate production and release from the intestine could be the source since it is the tissue with the highest accumulation of orally administered metformin (>100 times higher than plasma concentration) and the drug is shown to stimulate lactate production in the small intestine after in vivo metformin treatment of diabetic patients (Bailey, Wilcock, & Scarpello, 2008; Wilcock & Bailey, 1994).

The elevated exercise‐induced plasma adrenaline with metformin in the ST‐MES could indicate a greater whole‐body stress which also is supported by the higher RPE reported during exercise and a generally higher heart rate during the metformin ST‐MES exercise experimental day. A few studies have also reported increased HR (Boule et al., 2011) and RPE (Malin et al., 2010) while other missed a metformin effect during exercise on these parameters (Braun et al., 2008; Johnson et al., 2007). The reason for the inconsistency between these studies is not obvious but could be related to different combinations of metformin dose and duration as well as exercise duration and intensity. To our knowledge we are the first to report metformin‐ induced enhancement of adrenaline concentrations during exercise. In vitro cell studies and in vivo studies in rodents have indicated a stimulatory effect of catecholamines on AMPK activity in skeletal muscles and adipose tissue (Hutchinson & Bengtsson, 2006; Koh et al., 2007; Ruderman et al., 2003). Furthermore, Watt et al. (2006) reported increased adipose tissue AMPK activation in relation to exercise. However we have previously shown that catecholamines in humans do not influence AMPK activation in either skeletal muscle or adipose tissue during similar exercise to the present study (Kristensen et al., 2007). Thus we find it unlikely that the metformin‐induced enhancement of plasma adrenaline has influenced AMPK activation.

Taken together, short‐term metformin treatment, eliciting high plasma metformin concentrations, does not affect energy homeostasis and AMPK activation at rest or during exercise in skeletal muscle of healthy subjects. Importantly, our data, together with our recent study showing intact exercise‐induced AMPK activity in skeletal muscle from type 2 diabetic patients (Kjobsted et al., 2016), imply that patients on metformin medication likely have the same benefit of exercise/physical activity regarding AMPK regulation as healthy individuals. However, treatment with metformin is accompanied by enhanced perceived exertion and whole‐body stress during exercise. In perspective, this may provoke a lesser desire for physical activity in the metformin‐treated patients.
